# Biomechanical interplay between anisotropic re-organization of cells and the surrounding matrix underlies transition to invasive cancer spread

**DOI:** 10.1038/s41598-018-32010-3

**Published:** 2018-09-21

**Authors:** Deok-Ho Kim, Andrew J. Ewald, JinSeok Park, Kshitiz, Moonkyu Kwak, Ryan S. Gray, Chia-Yi Su, Jayhyun Seo, Steven S. An, Andre Levchenko

**Affiliations:** 1 Department of Bioengineering, University of Washington, Seattle, WA 98195 USA; 2 Department of Biomedical Engineering, Johns Hopkins University, Baltimore, MD 21218 USA; 3 Department of Cell Biology, Johns Hopkins University, Baltimore, MD 21218 USA; 4 Department of Biomedical Engineering, Yale University, New Haven, CT 06520 USA; 5 Yale Systems Biology Institute, Yale University, West Haven, CT 06516 USA; 6 School of Mechanical Engineering, Kyungpook National University, Daegu, 41566 Korea; 7 Department of Environmental Health and Engineering, Johns Hopkins Bloomberg School of Public Health, Baltimore, MD 21205 USA; 8 Department of Chemical and Biomolecular Engineering, Johns Hopkins Whiting School of Engineering, Baltimore, MD 21218 USA; 9 Department of Oncology, the Sidney Kimmel Comprehensive Cancer Center, Johns Hopkins School of Medicine, Baltimore, MD 21205 USA

## Abstract

The root cause of cancer mortality and morbidity is the metastatic spread of the primary tumor, but underlying mechanisms remain elusive. Here we investigate biomechanical interactions that may accompany invasive spread of melanoma cells. We find that metastatic cells can exert considerable traction forces and modify local collagen organization within a 3D matrix. When this re-organization is mimicked using a nano-fabricated model of aligned extracellular matrix fibers, metastatic cells, including less invasive melanoma cells, were in turn induced to align, elongate and migrate, guided by the local ridge orientations. Strikingly, we found that this aligned migration of melanoma cells was accompanied by long-range regulation of cytoskeletal remodeling that show anisotropic stiffening in the direction of fiber orientation suggestive of a positive feedback between ECM fiber alignment and forces exerted by cancer cells. Taken together, our findings suggest that the invasive spread of cancer cells can be defined by complex interplay with the surrounding matrix, during which they both modify the matrix and use the matrix alignment as a persistent migration cue, leading to more extensive and rapid invasive spread.

## Introduction

Living tissues can be seen as active materials with complex properties arising from the forces generated by individual cells constituting these tissues. These tissues can undergo substantial re-organization both normally, e.g., as a part of organism development, or abnormally, e.g., in human malignancies. The mechanisms governing such complex morphogenic events are still poorly understood, as are the fundamental laws governing the active material properties of such tissues. Understanding these properties can provide important clues to understanding complex human pathologies, including cancer metastasis.

A classical view of tumor metastasis is that this process begins with the acquisition of traits that allow malignant cells to escape from the primary tumor, to invade the local supporting tissue while interacting with extracellular matrix (ECM), ultimately entering the circulation^1–3^. Metastasis then progresses via transportation of cancer cells through blood circulation to distant sites, whereupon individual cells adhere, spread and migrate through ECM at the distant tissue and form secondary tumors^4,5^. This process can be particularly pronounced in aggressive tumors, including melanoma. Melanoma is the leading cause of death from skin cancer worldwide^6,7^. Morbidity and mortality in this cancer are attributable to the metastatic spread of primary tumors defined in turn by gene-environmental interaction^8^.

In this metastatic-invasive cascade, the abilities of cancer cells to invade ECM, to successfully navigate towards and away from blood vessels, and to withstand mechanical stress imposed by this migration, are enabled, in large part, by material properties of the cytoskeleton^9–11^. The cytoskeleton is a network of biopolymers within living cells that confers cell’s mechanical structure, as well as transmits physical forces to and from the ECM in the surrounding tissue microenvironment^12–14^. Intravital imaging of the tumor microenvironment during metastatic transition has revealed an altered stroma, with individual cancer cells and cell clusters migrating along highly aligned ECM fibers^15,16^. In addition, a growing number of studies have reported that cancer cell migration and invasion are correlated with an increased ability of malignant cells to exercise appreciable contractile force upon their surroundings^17–20^. Recently, high-frequency microrheology analysis revealed distinct mechanical features between benign and malignant cells^21^. These findings, taken together, underscore the importance of mechanical coupling between ECM and the cytoskeleton during cancer cell metastasis. However, the manner in which cytoskeletal dynamics and physical force transmission are correlated with metastatic potential, particularly within aggressive cancers, remains largely unexplored. Furthermore, the mechanical mechanisms by which cancer cells sense and respond to the alteration of ECM topography during their pilgrimage from the primary tumor site to distant organs remain to be fully elucidated.

To gain a better insight into the underlying mechanisms of these processes, one can benefit from decoupling the feedback between ECM and cytoskeletal reorganization, whose complexity can cloud the underlying mechanisms. One can separately explore how individual cells derived from tumors with different invasive capacity can deform the matrix, and how they can respond to a model matrix that has pre-defined and fixed organization. In this study, we followed this research approach taking particular advantage of a nano-fabricated ECM-coated cell adhesion substratum that mimics the fibrous, topographic features of the collagen matrix reorganized by active interaction between metastatic melanoma cells and surrounding matrix, with nano-scale resolution^22^. We showed that melanoma cells derived from tumors with different invasive and metastatic potential vary in their ability to both re-organize the surrounding matrix and respond to this re-organization as demonstrating phenotypically cancer invasiveness due to their distinct microrheology features.

## Results

### Melanoma cells with higher invasive potential exhibit stronger traction force and modify the organization of surrounding ECM

**Figure 1:**
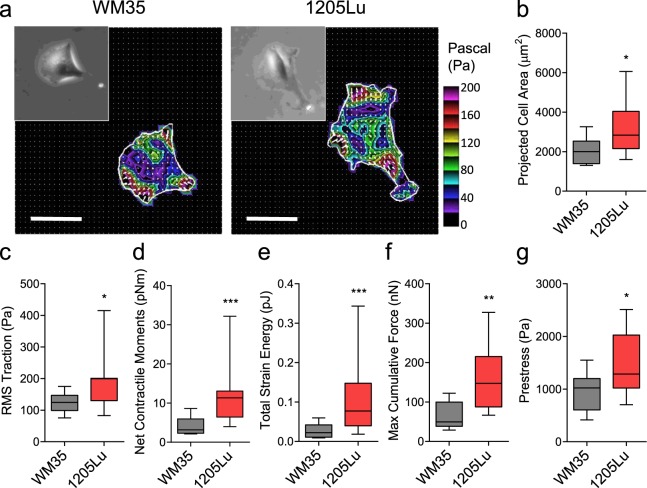
Less invasive (WM35) and highly invasive (1205Lu) cells exhibited different amounts of traction forces. (**a**) Representative traction maps of WM35 and 1205Lu cells measured with Fourier transform traction microscopy. Distributions of tractions are shown as vector maps and as color images after converting the magnitude of tractions into color codes as described. **(b)** Projected cell area, **(c)** RMS traction, (**d**) net contractile moment, **(e)** total strain energy, **(f)** maximum cumulative forces, and **(g)** prestress of WM35 and 1205Lu cells. (**b**–**g**) Box and whiskers plots, showing the median, the interquartile range, and the location of the minimum and maximum (n = 11 individual cell measurements per group). To satisfy the normal distribution assumptions associated with the Student’s t-test, data (RMS traction, net contractile moments and total strain energy) were converted to log scale prior to analyses. Scale bar: 50 µm in (**a**).

Mounting evidence suggests that tumor metastasis and, in particular, cancer cell migration and invasion require an appreciable exertion of contractile force upon the surrounding matrix^17,23^. Using Fourier transform traction microscopy, we first interrogated the force generating capacity of two melanoma cell lines occupying the opposite ends of an invasiveness spectrum^24,25^. Compared with less invasive WM35 cells, highly invasive 1205 Lu cells were appreciably bigger in size (Fig. 1a,b) and showed marked increases in traction (root mean square) average over the entire cell projected area (Fig. 1c). From the computed traction stress, we also derived a number of other metrics of intracellular forces, including the amplitude of net contractile moment (Fig. 1d), strain energy imparted by the cell to the substrate (Fig. 1e), maximum cumulative force (Fig. 1f), and the tensional stress borne by stress fibers (Fig. 1g). All computed physical metrics of forces were significantly greater in 1205 Lu cells than WM35 cells. In particular, the net contractile moment^26^, which is a scalar measure of the cell’s contractile strength, was 2.9-fold higher in 1205Lu cells than in WM35 cells (2.3407 ± 0.6114 vs. 1.2982 ± 0.5033, log transform Mean ± SD; P = 0.0003).

**Figure 2:**
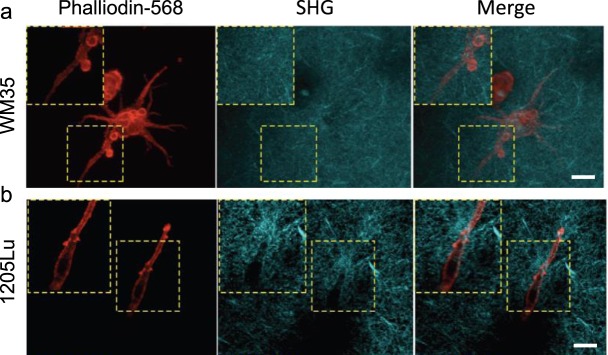
Highly invasive (1205Lu) cells remodeled surrounding matrix. Divergence of phenotypes in different melanoma cells within 3D collagen I cultures observed by second harmonic generation microscopy analysis. **(a)** WM35 cells exhibit a non-polarized spreading cell morphology (red arrowheads inset) and show no noticeable changes in the organization of collagen I fibers (SHG signal/cyan). **(b)** 1205Lu cells display a polarized spindle cell morphology in polarized organized collagen I fibers (SHG signal/cyan) adjacent to elongated 1205Lu cells. Scale bars: 30 µm.

Consistent with the mechanistic notion that increased cell traction force can distort and re-orient the ECM^27,28^, second harmonic imaging using two-photon microscopy of cells cultured inside a 3D collagen I matrix showed that, unlike WM35 cells, 1205Lu cells were able to rearrange the surrounding collagen matrix (Fig. 2a and b). Strikingly, the collagen ECM remodeled by highly invasive cells took on a form of long, fibrous micro-structures that ran parallel to the major axis of polarized cells, displaying characteristic migratory shapes. These findings support the concept of an increased force generating capacity of highly invasive melanoma cells and suggest a close mechanical coupling between traction force generated by the cells and the resultant local micro-configuration of the ECM. Our findings are also consistent with intravital imaging of the microenvironment in diverse tumors *in vivo*, showing highly polarized collagen matrix and the locomotion of individual cancer cells that tracks along such ECM structure^15^. However, it is not clear if, and to what extent, melanoma cells with different invasive abilities have different sensitivity to mechanical cues that might be present in the polarized collagen fibers and in integrating these signals into their migratory behaviors.

### Anisotropic nanotopography can guide cytoskeletal remodeling and the polarization of melanoma cells

**Figure 3:**
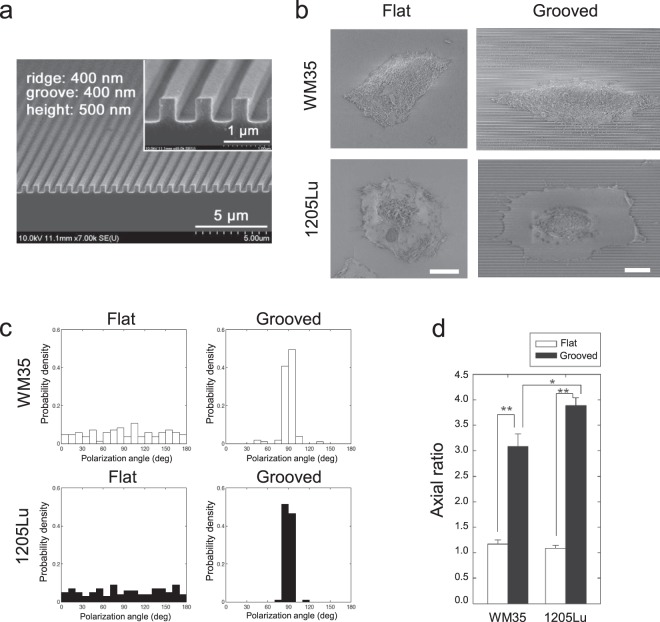
Enhanced polarization and elongation of both less and highly invasive melanoma cells guided by anisotropic nanotopography. (**a**) SEM image of a nanopatterned substratum with ridge = 400 nm, groove = 400 nm, and height = 500 nm; inset shows the enlarged image. **(b)** SEM images showing distinct morphological states of less invasive melanoma cells (WM35) and highly invasive melanoma cells (1205Lu) cultured on the flat surface and the grooved substrata. **(c)** Polarization angle distribution of cell morphology in WM35 and 1205Lu cells cultured on flat, and grooved substrata. **(d)** Morphological changes in both non-metastatic and metastatic melanoma cells guided by the grooved substrata, described by axial ratio of *L*_y_/*L*_x_. Error bars represent mean ± SEM. Scale bar: 10 µm in (**b**).

One way to decouple the feedback between the ability of cells to deform the matrix and the ability of the deformed matrix to define the cells’ behavior is to independently control one aspect of this feedback, e.g., the organization of the matrix fibers. To achieve this, while simultaneously retaining the ease of analysis associated with 2D cultures, we used an experimental platform based on nanofabricated arrays of parallel fibers separated by ‘grooves’ mimicking in dimension the sizes arrays of large aligned collagen fibers^29^. Previously, we and others^30–33^ have shown that cells on such nano-scale nano-fiber/groove arrays are highly responsive to the mechanical cues provided by the orientation of grooves and their sizes, in ways highly reminiscent of individual and collective cell behavior in 3D. The control of the fiber-like ridge orientation allows one to examine in particular whether the non-invasive and metastatic melanoma cells would be equally sensitive to the mechanical cues that might arise from oriented collagen matrix. In this study, the feature sizes of the ECM-mimicking ridge arrays were 400 nm_400 nm_500 nm (nano-fiber width_groove width_height) (Fig. 3a), similar to the range of widths of collagen fibers in native ECM surrounding *in vivo* tumors^34,35^.

On the flat surface, both highly invasive (1205Lu) and less invasive (WM35) melanoma cells displayed rounded cell morphology (Fig. 3b). In contrast, melanoma cells, including even less invasive cells that have the limited potential to remodel ECM fibers, cultured on the grooved surface were spindle-like in morphology and were elongated along the direction of grooves (Fig. 3b–d). Cell polarization angle was uniformly distributed in cells adhering to the smooth surface, whereas it was narrowly distributed in cells adhering to grooved surface (Fig. 3c). Cells adhering to the grooved surface also showed a marked increase in cell elongation, as measured by their axial ratio (Fig. 3d). Cell polarization and elongation along the direction of grooves were more prominent for the highly invasive 1205Lu cells vs. less invasive WM35 cells (Fig. 3c,d).

**Figure 4:**
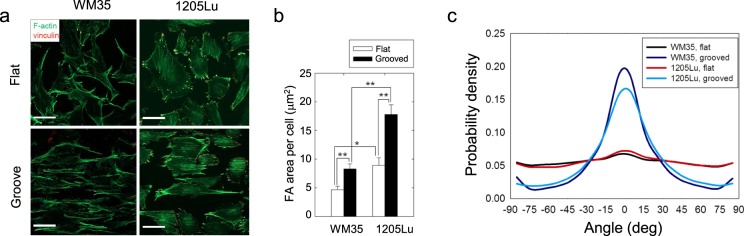
Anisotropic arrangement of ventral cytoskeleton and increased integrin expression in 1205Lu cells cultured on nanogrooved substrata. (**a**) Representative immunofluorescent micrograph showing the spatial distribution of actin stress fibers (green) and vinculin (red) of non-metastatic and metastatic melanoma cells cultured on the flat and the grooved substrata. (**b**) Quantitation of total FA area per cell, for WM35 cells and 1205Lu cells cultured on the flat surface and the grooved substrata. (**c**) Probability density of correlation of the direction of actin fibers aligning with the direction of grooves (0 deg refers to parallel direction to the grooves). Error bars represent mean ± SEM. Scale bar: 30 μm in (**a**).

Pronounced changes in cell morphology on the grooved surfaces suggest marked changes in cytoskeletal organization. We thus assessed structural organization of actin microfilaments and focal adhesion protein, vinculin, within both melanoma cell lines. Consistent with the random distribution of cell polarization angle on the flat surface (Fig. 3e), actin microfilaments were randomly distributed in both WM35 and 1205Lu cells cultured on the flat, smooth surface (Fig. 4a, top panels). By contrast, both WM35 and 1205Lu cells cultured on the grooved surface showed highly oriented actin microfilaments aligned in the direction of the grooves (Fig. 4a, bottom panels). Furthermore, cells also displayed elevated formation of focal adhesions (FA) on the grooved surface, showing pronounced patterns of vinculin bands also oriented in the direction of grooves (Fig. 4a,b). Overall, in the presence of nanotopographic cues, the stress fiber network displayed an almost perfect alignment along the direction of grooves, suggesting that both cell migration directionality and force application would be anisotropically enhanced in the direction of the ECM-like ridges (Fig. 4c).

### Oriented ECM-like ridge arrays impose anisotropic stiffening of cytoskeleton

Our data suggested that the cytoskeletal network could undergo extensive reorganization and polarization in the direction of the nanogroove arrays on the cell adhesion substratum. As a consequence, we set out to explore whether this anisotropic cytoskeleton orientation would also lead to corresponding anisotropic changes in cell stiffness.

**Figure 5:**
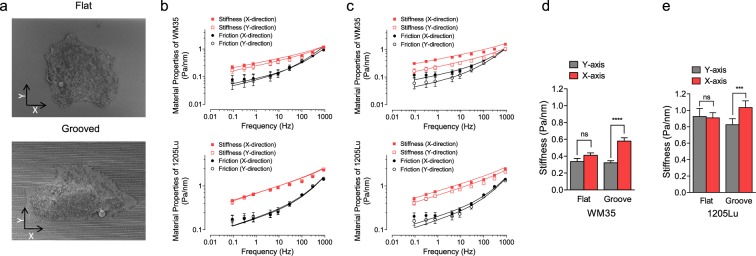
Melanoma cells exhibit anisotropic stiffening in the direction of cell migration. (**a**) Electron micrographs of the cells and attached beads on the cell membrane on flat and grooved substrata. X-axis refers to direction in parallel to grooves, and Y axis refers to the direction perpendicular to the grooves. (**b**,**c**) Material properties (stiffness and friction moduli) measured by Magnetic Twisting Cytometry over five decades of probing frequencies in cells cultured on flat (**b**) and nano-grooved (**c**) substrata. Data are presented as Geometric Mean ± SE (n = 254–940 individual cells on flat; n = 400–784 individual cells on groove). The solid lines are the fit of experimental data to the structural damping equation with addition of a Newtonian viscous term as previously described^55^. (**d**,**e**) Stiffness measured at 10 Hz, showing anisotropic stiffening of WM35 and 1205Lu cells on the grooves.

To accomplish this, we examined the forced motions of magnetic field-driven micro-beads functionalized to the cortical cytoskeleton through the cell surface integrin receptors expressed in melanoma cells^36,37^ (Fig. 5a). we measured the cytoskeletal stiffness (measured as the elastic or storage modulus, *g*′) and internal energy dissipation (estimated as the loss modulus, *g*″) of the melanoma cell lines using magnetic twisting cytometry^38^, in cells cultured on flat or grooved surfaces. We found that, on both flat and grooved surfaces, the stiffness of both WM35 and 1205Lu cells increased with frequency as a weak power law (Fig. 5b,c). Friction also followed a weak power law at lower frequencies (below ~10 Hz), but showed a stronger frequency dependence at higher frequencies (Fig. 5b,c). We found that *g*′ and *g*″ of both cell lines were isotropic (equal for magnetic bead displacement both along and perpendicular to a randomly chosen direction), if cells were cultured on the flat surfaces. In striking contrast, *g*′ and *g*″ for both cell lines were highly anisotropic with respect to the direction of the grooves on the nano-structured surfaces. Accordingly, melanoma cells exhibited anisotropic stiffening and viscous cytosol flow in the direction of cell migration. Our findings hence suggest that the actomyosin networks defining the apparent cell stiffness are differentially regulated along the nan-fiber/groove direction, suggesting that the anisotropic cues arising from the surface nano-topography propagate throughout cortical cytoskeleton, reflecting of extensive re-organization of the cytoskeleton. This cytoskeleton re-organization can lead to anisotropic force generation, which can result in both polarized force application and enhanced polarization of the locomotion apparatus, something we addressed next.

### Anisotropic nanotopography guided the directional migration of melanoma cells

**Figure 6:**
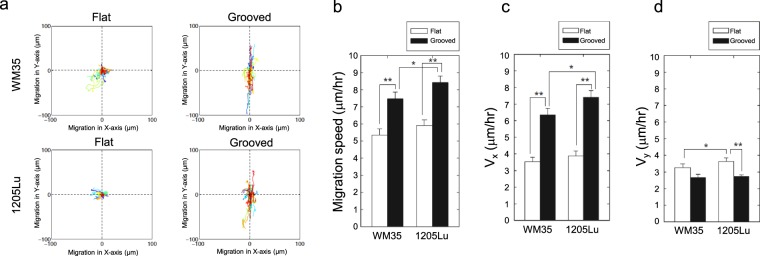
Nanotopography enhances the migration of melanoma cells along nanotopograpy. (**a**) Effect of anisotropic topography stimulation on cell migration tracks of WM35 and 1205Lu cells. (**b**) Migration speed of WM35 and 1205Lu cells cultured on the grooved substrata and flat substrata. (**c**,**d**) Migration speed of WM35 and 1205Lu cells (**c**) parallel to the direction of grooves and (**d**) perpendicular to the direction of grooves.

The results so far suggest that more invasive melanoma cells can align the ECM fibers and melanoma cells with different degrees of invasiveness can respond to this alignment by altering their shape and cytoskeletal structure and dynamics in a highly anisotropic fashion. A critical question is whether these anisotropic, polarized changes in the dynamic cytoskeleton can translate into anisotropic cell motility. We found that both highly invasive and less invasive melanoma cells cultured on the grooved surface displayed more extensive cell migration relative to the flat surface (Fig. 6a,b). Whereas cell migration on the flat surface was random and limited in extent, motility on the grooved surface was anisotropic and strongly biased in the direction of the grooves (Fig. 6a). For both cell lines, in the direction of the grooves, cell migration speed was approximately 2-fold faster than the corresponding speed values achieved on the flat surface (Fig. 6c,d). Interestingly, the speed of migration of both cell lines in the direction perpendicular to the grooves was not only several-fold lower than along the groove direction, it was also significantly lower than the speed on the flat surfaces. Furthermore, highly invasive 1205Lu cells moved significantly faster than less invasive WM35 cells, particularly in the direction of grooves (Fig. 5b–d). This result suggests that nanotopographic features of local ECM, independent of other guidance factors, may strongly influence both direction and the magnitude of cell migration, with the effect further modulated by genetically defined invasive potential.

## Discussion

Progression of melanoma from the relatively benign, radial growth phase, to the much more aggressive and invasive, vertical growth phage is associated with a worsening prognosis for the patients, in part due to an increased probability of metastatic spread^39,40^. The molecular and phenotypic determinants of this transition are under intense scrutiny. Here, we provide evidence that the invasive and metastatic state can be associated with distinct mechanical cell characteristics, particularly contractility. This alteration can have important consequences for cell interaction with the surrounding matrix, composed mostly of collagen *in vivo*. First, there is a greater ability of the aggressive and metastatic cells to restructure the matrix, creating aligned fibers, which can then be interpreted by both highly invasive and less invasive cells as a contact guidance cue directing their migration. Thus, the material properties of the combination of cells and matrix can change considerably. Interestingly, the mechanical properties of melanoma cells interacting with aligned nano-scale fibers display further alterations, with the cytoskeletal alignment and cell stiffness increasing in the direction of the fibers, but not in the orthogonal direction. These alterations suggest that the ability of the cytoskeleton to apply force on the surrounding ECM can itself be polarized, as the polarization of ECM increases.

These findings support existence of a positive feedback between the fiber alignment and the ability of the cells to deform the fibers further. Additionally, an increased anisotropy of the cytoskeleton can enhance polarization of the locomotion apparatus, enhancing the persistence of cell migration and accounting for the contact guidance observed in our experiments. Furthermore, the anisotropic stiffness of the cells migrating along the nano-fabricated ridges or ECM fibers suggests the ability to better resist the shearing forces induced by the migration of the cell itself. Indeed, as the cells migrate, the connections to the matrix can both provide the cell with the necessary points for locomotive force application, but also create the points of resistance to overall cell displacement. Interestingly, a similar adaptive emergence of anisotropic stiffness has been observed in endothelial cells resisting shear flow^41^, suggesting the importance of this mechanism for adaptation to anisotropic changes in the material properties of the surrounding medium.

Re-organization of ECM resulting in its alignment has been suggested before as an important precursor to metastatic spread in different cancer types^16,42^, including breast cancer^43^, glioblastoma^44,45^, and pancreatic cancer^46^. To enable this change, the constituent cells need to be able to exercise sufficient forces, the capability usually associated with fibroblasts, smooth muscle or myoepithelial cells. Analysis of alignment of ECM fibers by fibroblasts has a long history. Of interest, it has been suggested that squamous carcinoma cells can recruit fibroblasts to enable matrix re-organization and subsequent collective directed migration^47^. We find that invasive melanoma cells can acquire the ability to deform and align the surrounding matrix, which obviates the need for recruitment of other cell types and thus enables the forthcoming migration and invasion.

The results presented here suggest that the anisotropic cues arising from the re-organized nanotopography of the cell microenvironment coupled with the intrinsic mechanical characteristics of cancer cells embedded in this microenvironment, can alter the invasive properties of tumors, in a way dependent on the regulation of the cell cytoskeleton. The sensing of the mechanical cues provided by the aligned ECM fibers is still subject to intense research and may involve the topographic cues, as suggested by our recent analysis of melanoma cells^25,48^ and/or sensing of the anisotropic nature of the adhesion substratum, as nanofiber arrays are effectively stiffer along their orientation and less stiff in the orthogonal direction. Larger and denser aligned ECM fiber clusters might also be perceived by tumor cells as adhesion substrata that would be similar to micro-contact printed ECM stripes, which, as observed previously^49^, might also contribute to cytoskeleton re-organization and directed cell migration. Whatever the mechanism however, the ensuing cytoskeleton re-organization would feed back onto the anisotropic ECM structure, closing the feedback.

Overall, our results further suggest that understanding such complex phenotypes of cancerous cells and tissues, as invasive growth, require more complex context of the interaction between the cell and the surrounding environment, whose organization and mechanical properties can be strongly affected by and in turn affect the properties of the embedded cells. Moreover, our findings suggest that targeting the biomechanical interaction between cancer cells and their microenvironment may lead to promising therapeutic strategies.

## Methods

### Cell culture

Melanoma cell lines 1205Lu and WM35 were cultured in Dulbecco’s modified Eagle’s medium (Gibco) supplemented with 10% fetal bovine serum (Gibco), 50 U/ml penicillin, and 50 μg/ml streptomycin (Invitrogen) at 37 °C, 5% CO_2_ and 90% humidity. These were split 1:4 after trypsinization every 2–3 days. A glass coverslip with the topographical pattern substratum was glued onto the bottom surface of the custom-made MatTek dish (P35G-20-C). Then, we replated cells on the patterned substratum pre-coated by 50 μg/ml of fibronectin for 3 h and incubate up to 12 h for the attachment.

### Preparation of nanopatterned cell culture substrata

The soft poly(urethaneacrylate) (PUA) mold material consists of a functionalized precursor with an acrylate group for cross-linking, a monomeric modulator, a photoinitiator and a radiation-curable releasing agent for surface activity^50^. Details of the synthesis and characterization of the material have been published elsewhere. To fabricate a sheet-type mold, the liquid mixture was drop-dispensed onto a silicon master pattern and then a flexible, transparent polyethylene terephthalate (PET) film was brought into contact with the liquid mixture. Subsequently, it was exposed to UV light. After UV curing, the mold was peeled off from the master pattern and additionally cured overnight to terminate the remaining active acrylate groups on the surface. The resulting PUA mold used in the experiment was a thin sheet with a thickness of ~50 μm.

The polymeric nanostructures with periodic ridges and grooves were fabricated onto the glass coverslip using UV-assisted capillary lithography^30,31,51^. Prior to application of the PUA mold, the glass substrate was thoroughly rinsed with ethanol to remove excess organic molecules and dried in a stream of nitrogen. A small amount of the same PUA precursor was drop-dispensed on the substrate and a PUA mold was directly placed onto the surface. The PUA precursor spontaneously moved into the cavity of the mold by means of capillary action and was cured by exposure to UV (λ ~ 400 nm) for ~20 s through the transparent backplane (dose = 100 mJ/cm^2^). After the curing, the mold was peeled off from the substrate using sharp tweezers. The adhesion between the PUA nanopatterns and the glass substrate was promoted by spin-coating a thin layer (~ 200 nm) of the primer solution prior to dispensing of the precursor solution. As a control surface, we created a flat surface with same PUA. Instead of placing on a PUA mold, we put a transparent and flat PDMS block and repeated same procedure.

### Immunofluorescent microscopy

Melanoma cells were seeded onto the flat surface and the nanogrooved substrata and incubated overnight for 20 h. Cells were then fixed in 4% paraformaldehyde solution (Electron Microscopy Systems, Hatfield, PA) for 15 min, washed, and made permeably with Triton X-100 (0.1% v/v) in phosphate buffered saline (PBS) for 15 min, and then incubated for 1100 (0.1Alexa fluor 488 conjugated phalloidin (Invitrogen) and vinculin antibody as a primary antibody to stain for actin filaments and vinculin respectively. Samples were then further washed in PBS and sterile water before being treated with anti-fade reagent. The stained cells were imaged using a Zeiss LSM 510 Meta CLSM under 100 × and 63 × plan apochromatic objective lens 1.4 N.A. Images were processed using LSM 510 Zeiss Meta software version 3.5.

### Quantitative analysis of cell shape and migration

The custom-made Matlab script was used to identify cell boundaries from phase-contrasted images and measure cell centroid position. Average individual cell speeds (S) were calculated from individual cell tracks from the time series images taken every 10 minute by averaging the speeds measured every time interval, 10 minute. Because cell-cell contact is known to affect the extent of cell spreading and migration, only those cells that spread without contact with adjacent cells were analyzed. The axial ratio was defined as the ratio of the maximal cell cross-section length along the nano-grooves to that across the grooves (longitudinal vs. transverse directions). For each condition, over 40 cells were quantified in total. Student t-test was used to evaluate the statistical significance where indicated.

For quantitative analysis of cell orientation, we cultured WM35 and 1205Lu cells on the topographic substratum. Orientation angle of polarized cell was determined by measuring the acute angle between the major axis of the cell and the direction of grooves. A total of 40 cells for each group were used to construct the polarization angle distributions with range −90° and 90°. Positive and negative angles were defined to be counter-clockwise and clockwise direction, respectively. An angle of 0° was defined as the angle when cells were perfectly aligned parallel to the ridge/groove pattern arrays.

### Orientation analysis of F-actin and vinculin using pixel gradient algorithm

Fluorescently labeled phalloidin and vinculin stained images were used to detect the orientation of actin and focal adhesions. Orientation analysis was performed using the gradient orientation approach implemented in Matlab (Mathworks, Natick, MA) as previously described^52^. Briefly, for pixel gradient analysis, Gaussian low pass filter and Sobel horizontal edge-emphasize filter (predefined in Matlab Image Analysis Toolbox) were used to implement a two dimensional convolution. After transposing the Sobel filter to extract the vertical edges, both the horizontal and vertical edges were combined to calculate the gradient magnitude of each pixel in the image. Followed by thresholding the gradient magnitude to determine the contours of the area of interest, the gradient orientation was calculated by determining the angle of the gradient with respect to the x-axis.

### Fourier transform traction microscopy

The contractile stress arising at the interface between each adherent cell and its substrate was measured with traction microscopy^26,53,54^. Cells were plated sparsely on polyacrylamide elastic gel blocks (Young’s modulus of 8 kPa) coated with collagen type I (0.2 μg/ml) and traction was computed using Fourier transform traction cytometry as described previously^53,54^. The computed traction field was used to obtain net contractile moment, which is a scalar measure of the cell’s contractile strength^26^. Net contractile moment is expressed in units of pico-Newton meters (pNm). In brief, cells were plated sparsely on elastic gel blocks (~1,500 cells per gel block) coated with collagen type I, and allowed to adhere and stabilize for 24 h. For each adherent cell, images of fluorescent microbeads (0.2 μm in diameter, Molecular Probes, Eugene, OR) embedded near the gel apical surface was taken at different times; the fluorescent image of the same region of the gel after cell detachment with trypsin was used as the reference (traction-free) image. The displacement field between a pair of images was then obtained by identifying the coordinates of the peak of the cross-correlation function.

### Optical magnetic twisting cytometry (OMTC)

Stiffness of the living adherent cell was measured as described previously^53,55^. In brief, an RGD-coated ferrimagnetic described previously and then twisted in a vertically aligned homogenous magnetic field that varied sinusoidally in time. The sinusoidal twisting magnetic field causes both a rotation and a pivoting displacement of the bead: as the bead moves, the cell develops internal stresses which in turn resist bead motions^55^. Here we defined the ratio of specific oscillatory torque on the bead to lateral bead displacements as the complex elastic modulus G* = G′ + iG″ (spatial resolution being ~5 nm).

### 3D cell culture and second-harmonic imaging of collagen

Sterile Collagen I solution (BD Biosciences 354236) was pH adjusted, on ice, using 1 N NaOH yielding a final concentration of 1.5 mg/mL collagen I and 1 × DMEM. Individual melanoma cell lines were resuspended [1000 cells/μL] and plated as 100 μL gels on non-tissue culture treated 24-well plates to facilitate gel adherence. Gels were allowed to incubate at 37 °C in 5% CO_2_ for 30 min before standard media addition. After 48 h, gels were fixed in a 4% PFA in 2% sucrose solution for 3 h at room temperature. Alexa-Fluro-568 conjugated Phalloidin (Invitrogen A12380) was used to visualize the cell cortex (F-actin). Confocal imaging and Second Harmonic Generation (SHG) of collagen I fibers were done sequentially using a Zeiss LSM 710NLO-Meta using a 40×/1.1 W LD C-Apo objective. Standard confocal imaging of (1 Airy unit and 5% laser power) was utilized for collection of the Phalliodin-568 signal, while the SHG signal was detected using the maximum adjusted pinhole and 40% laser power. Images were overlaid and equally adjusted for minimum and maximum intensity using IMARIS (Bitplane).
